# Functionally Enigmatic Genes in Cancer: Using TCGA Data to Map the Limitations of Annotations

**DOI:** 10.1038/s41598-020-60456-x

**Published:** 2020-03-05

**Authors:** Alexandra Maertens, Vy H. Tran, Mikhail Maertens, Andre Kleensang, Thomas H. Luechtefeld, Thomas Hartung, Channing J. Paller

**Affiliations:** 1 Johns Hopkins University, Bloomberg School of Public Health, Center for Alternatives to Animal Testing (CAAT), Baltimore, MD United States; 2 ToxTrack LLC, Baltimore, USA; 3 University of Konstanz, CAAT-Europe, Konstanz, Germany; 4 Johns Hopkins University, Oncology, Baltimore, USA

**Keywords:** Cancer genomics, Gene ontology

## Abstract

Cancer is a comparatively well-studied disease, yet despite decades of intense focus, we demonstrate here using data from The Cancer Genome Atlas that a substantial number of genes implicated in cancer are relatively poorly studied. Those genes will likely be missed by any data analysis pipeline, such as enrichment analysis, that depends exclusively on annotations for understanding biological function. There is no indication that the amount of research - indicated by number of publications - is correlated with any objective metric of gene significance. Moreover, these genes are not missing at random but reflect that our information about genes is gathered in a biased manner: poorly studied genes are more likely to be primate-specific and less likely to have a Mendelian inheritance pattern, and they tend to cluster in some biological processes and not others. While this likely reflects both technological limitations as well as the fact that well-known genes tend to gather more interest from the research community, in the absence of a concerted effort to study genes in an unbiased way, many genes (and biological processes) will remain opaque.

## Introduction

In the years since the Human Genome Project was completed, our understanding of the function of genes has grown by leaps and bounds. In 1998, barely 15 percent of the human genome had been sequenced; by 2000, a working draft of the genome had been completed, as sequencing technology increased in speed and dropped in cost^1^. Today, sequencing is sufficiently inexpensive and rapid that researchers have at their disposal thousands of tumor tissues with RNA-Seq data, providing unprecedented insight into the transcriptional landscape of cancer^2,3^.

However, the sheer volume of data has proven challenging when it comes to deriving biological meaning. Many types of analysis, such as over-representation analysis, gene set enrichment analysis (GSEA)^4^, signaling pathway impact analysis^5^, and pathway-specific analysis (PARADIGM)^6^, all rely to some degree on *a priori* knowledge of the pathways, the biological role, or the molecular function of genes in order to identify meaningful patterns in the data. The most common databases, Gene Ontology (GO) database^7^, and Kyoto Encyclopedia of Genes and Genomes (KEGG) pathway database^8^, provide annotations that allow researchers to analyze high-throughput data to focus on biological processes and look at the functional output of a system, rather than focus on an individual gene. As such, annotations have been key to improving the statistical power of transcriptomic studies by alleviating the multiple-hypothesis testing problem of transcriptomic data and providing a higher-level view of coordinated biological processes^9^. For example, pathway-based approaches have proven critical for understanding molecular signatures of colon cancer^10^ as alterations in the Wnt signaling pathway were identified as common in colorectal cancers, regardless of whether the tumor was hypermutated or non-hypermutated.

Despite these advantages, annotation-based approaches have some significant drawbacks that limit the insights that can be gleaned from any analysis that relies exclusively on annotations. For instance, GO ontology was developed as an attempt to unify vocabulary from the terms developed for model organisms – Fly (*Drosophila melanogaster*), Mouse (*Mus musculus*), and Yeast (*Saccharomyces cerevisiae*)^7^– and there is a strong underlying assumption that orthologous genes share similar biological functions^10^. While this is often a safe assumption, this approach may overemphasize highly conserved cellular processes and potentially overlooks important species-specific and/or tissue-specific functions. Additionally, the error rate of curated eukaryotic sequence annotations is not trivial – one study found an error rate of between 33% and 43% when comparing UniProt to SwissProt database annotations^11^. The GO term annotation error rate estimates 13% to 18% for curated annotations in the GoSeqLite database, 49% for annotations inferred from sequence similarity, and 28% to 30% for all curated annotations^12,13^. Finally, the information that is missing from annotation-based approaches is not missing at random. Genes that are present in the databases assembled for specific purposes (e.g. Cytogenetics in Oncology and Haematology (ATLAS), Human Gene Mutation (HGMD), Drug Response Variations (PHARMGKB), etc.) tend to have significantly more annotations than other genes, as do protein-coding genes in general compared to non-coding RNA^14^. Therefore, relying on annotations alone for a complete functional understanding of any given experiment may, in the best-case scenario, unnecessarily limit what can be seen, while in the worst-case scenario is equivalent to looking under the lamppost for the lost keys, since that is where the light is.

This paper draws attention to the fact that a substantial portion of genes statistically associated with cancer biology lack annotations adequate for understanding their role in cancer pathology. We refer to these genes as “functionally enigmatic genes” (adopted from a previous study focusing on neuroscience^15^). We show that there is no indication that the bibliometric attention paid to genes in any way correlates with their role in cancer when measured either by network topology of gene importance or by strength of association with clinical outcome; that these genes are not distributed evenly throughout the network; and that these genes are not likely missing at random, but rather represent blind spots in our map of gene interactions and functions.

## Results

### Most genes associated clinically with cancer have a minimal literature base and inadequate pathway annotations

**Figure 1:**
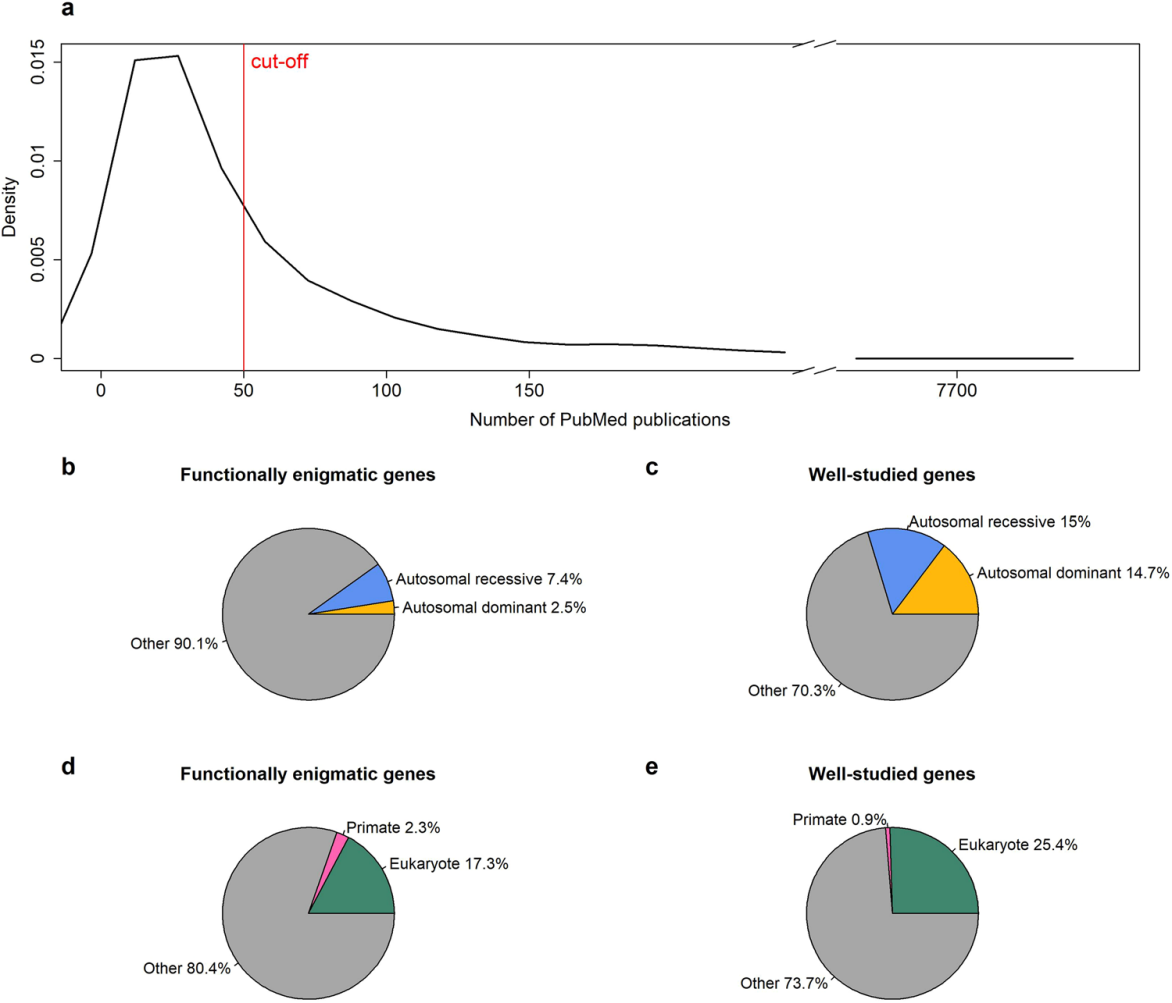
(**a)** Density of PubMed IDs (PMIDs) per gene for all prognostic unfavorable genes in various types of cancer from the Human Protein Atlas; the bulk of genes have few articles and the density begins to decrease sharply at 100. Genes with ≤50 PMIDs are defined as functionally enigmatic genes; and genes with number of PMIDs >50 are considered well-studied genes. (**b**,**c)** Distribution of autosomal dominant and recessive disease associations among functionally enigmatic genes and well-studied genes. (**d**,**e)** Distribution of primate-specific genes and conserved eukaryotic genes among functionally enigmatic genes and well-studied genes.

We began by exploring genes associated with an unfavorable outcome in cancer in the Human Protein Atlas Pathology Atlas, which contains a correlation of mRNA and clinical outcome for almost 8,000 cancer patients with a *p-*value <0.001; this generated over 6,744 genes with some clinical significance to an unfavorable outcome in cancer. We next counted the PMIDs per gene, determined by querying Entrez ID of each gene in the PubMed database. We classified any gene with less than 50 PMIDs as a cut-off “functionally enigmatic,” as this likely represents a level at which the literature base is inadequate to fully understand gene function. By this rough metric, over 4,537 genes are inadequately annotated compared to 2,207 genes with more than 50 PMIDs (Fig. 1a).

We focused our attention on genes with a significant association with clinical outcomes. Such genes would provide less information when studied using any approach (such as enrichment analysis) that relies upon annotations. While over ten percent of the functionally enigmatic genes were “unclassified” in GO Annotations for Biological Process, less than one percent were for annotated genes. However, this likely overstates the informativeness of the GO annotations for the functionally enigmatic genes, as many of the available annotations were relatively high-level [e.g. “cellular metabolic process” (GO:0044237)] and would not necessarily be informative for data analysis. Using Panther GO Slim - which significantly pares down the GO annotations to eliminate redundancy and focus on the most informative and evolutionarily conserved annotations - half the functionally enigmatic genes were unclassified *vs*. one-third of the “well-annotated” genes. More drastically, of the functionally enigmatic genes, 4,224 of the 4,527 genes were not on a PANTHER Pathway, compared to 1,507 of the 2,207 well-studied genes (Table 1). Therefore, the understudied genes represent a substantial proportion of the biological signal that would be invisible in many annotation approaches, and in terms of a robust understanding of the likely pathways, for most genes this information is lacking.

**Table 1:** Comparison of available annotations for Functionally enigmatic genes *vs*. well-studied genes.

	Functionally Enigmatic	Well-studied
GO Unclassified	10.8%	<0.05%
GO Slim Unclassified	53%	32%
Panther Pathways Unclassified	93%	68%

Functionally enigmatic genes were more likely to be unclassified in GO, the narrower and precise annotations in GO Slim, as well as Panther Pathways. All differences were significant (p value <0.05) by a chi-square test.

### Functionally enigmatic genes are less likely to be classified as associated with an autosomal dominant or recessive inherited disease, and more likely to be primate-specific

One key question is why such genes are missing, and equally important: are they missing at random, or is there a bias that explains their relative absence in the literature? Not surprisingly, the functionally enigmatic genes were less likely to be conserved in eukaryotes and more likely to be primate specific (*homo sapiens*, *homo* and *catarhini)* (Fig. 1b,c; See Supplementary Table 1 for a full listing of lineage classifications). Additionally, genes that were classified with either “autosomal dominant” or “autosomal recessive” associations with diseases by the Human Phenotype Ontology were over-represented among the well-studied genes – combined, they constituted almost 30 percent of the well-studied genes, compared with 10 percent of the functionally enigmatic genes (Fig. 1d,e). While this bias likely reflects the fact that many genes were initially discovered because they caused a clearly observable phenotype, it is still surprising, given that polygenic diseases occur much more frequently and with greater societal impact^16^. Although the last decade has seen an extensive genome-wide association studies (GWAS) literature-based mining for associations, the literature is likely biased by the challenge of studying the molecular mechanisms of genes that each contribute in a small way to an observable pathology and by the fact that researchers may simply favor genes with a clear mechanistic association with a disease.

### Genes are not studied in proportion to their importance in network topology or clinical significance

As the Human Protein Atlas focused only on genes associated with survival, and therefore is skewed towards more lethal cancers such as renal cancer, we expanded our approach to three types of cancer [glioma (GBMLGG), colon cancer (COAD), and prostate cancer (PRAD)] using different ways of selecting genes with clinical significance: for glioma, we chose genes with a correlation with “days to death” with a *q*-value <0.01 and C-Index greater than 0.6; for prostate cancer, we chose genes correlated +/− with Gleason score with a *q*-value <0.01, and for colorectal cancer, we chose genes from a landmark TCGA study^17^, that calculates the aggressiveness score as a composite of association score with six clinical variables using the weighted Fisher’s method, from which an overall combined *p*-value is derived, reported as the negative of the base-10 logarithm, and assigned a plus or minus depending on whether the signature is higher or lower in the more aggressive tumors, with a cut-off of +/− 3 (equivalent to an uncorrected a *p-*value of 0.001).

Since one key feature of cancer is uncontrolled transcription, many of these genes are likely statistically significant but not biologically significant - in other words, the correlation with phenotype might be incidental rather than reflecting a gene that is driving a biologically significant outcome. Therefore, in order to focus on biologically relevant genes, we created a weighted gene correlation network using the weighted correlation network analysis (WGCNA) package^18^ for each dataset from the available RNASeq data, after filtering the most variant 10,000 genes based on expression levels. That preferentially eliminated many of the genes classified as functionally enigmatic, but left a proportion of genes similar to the analysis based on the Human Protein Atlas (i.e., approximately 70 percent classified as functionally enigmatic, *vs* 78 percent for all the genes called based on GDAC Firehose analysis). WGCNA is essentially a “guilt-by-association approach” that uses a network topology metric to improve feature selection; this approach allowed us to focus on genes for which there was evidence at the network level that they were biologically significant, in addition to the metric of clinical significance.

**Figure 2:**
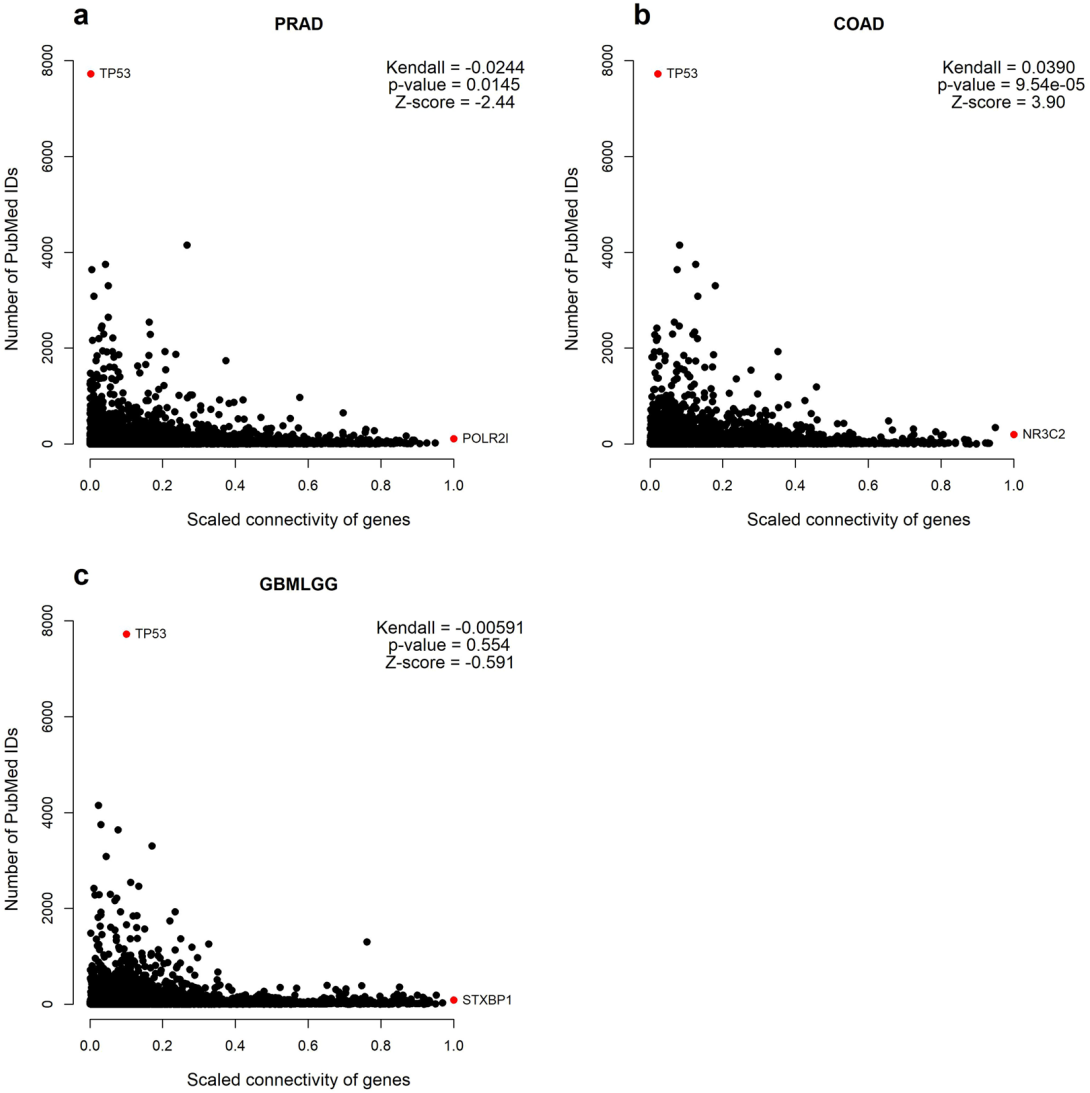
Kendall correlation between scaled connectivity and number of PubMed publications in different cancers in (**a)** prostate adenocarcinoma dataset (PRAD) **(b)** colon adenocarcinoma (COAD) and (**c**) glioma (GBMLGG). Highlighted genes (red) include outliers in terms of publication as well as the functionally enigmatic gene with the highest ranking scaled-connectivity.

For each data set, we examined whether scaled connectivity (a metric of whether a gene is acting as a “hub”) correlated with the depth of literature base using Kendall rank correlation; in the PRAD dataset, the correlation was miniscule, negative, and barely statistically significant; in the COAD data set, the correlation was miniscule, positive, and statistically significant, and the correlation was insignificant in the GBMLGG dataset (Fig. 2a–c). In all likelihood the variation is due to a few outliers (i.e. TP53), that have a disproportionate number of PMIDs. In aggregate, our data suggest that by the metric of scaled connectivity, there is minimal reason to believe that research efforts are focused on the most pertinent genes.

**Figure 3:**
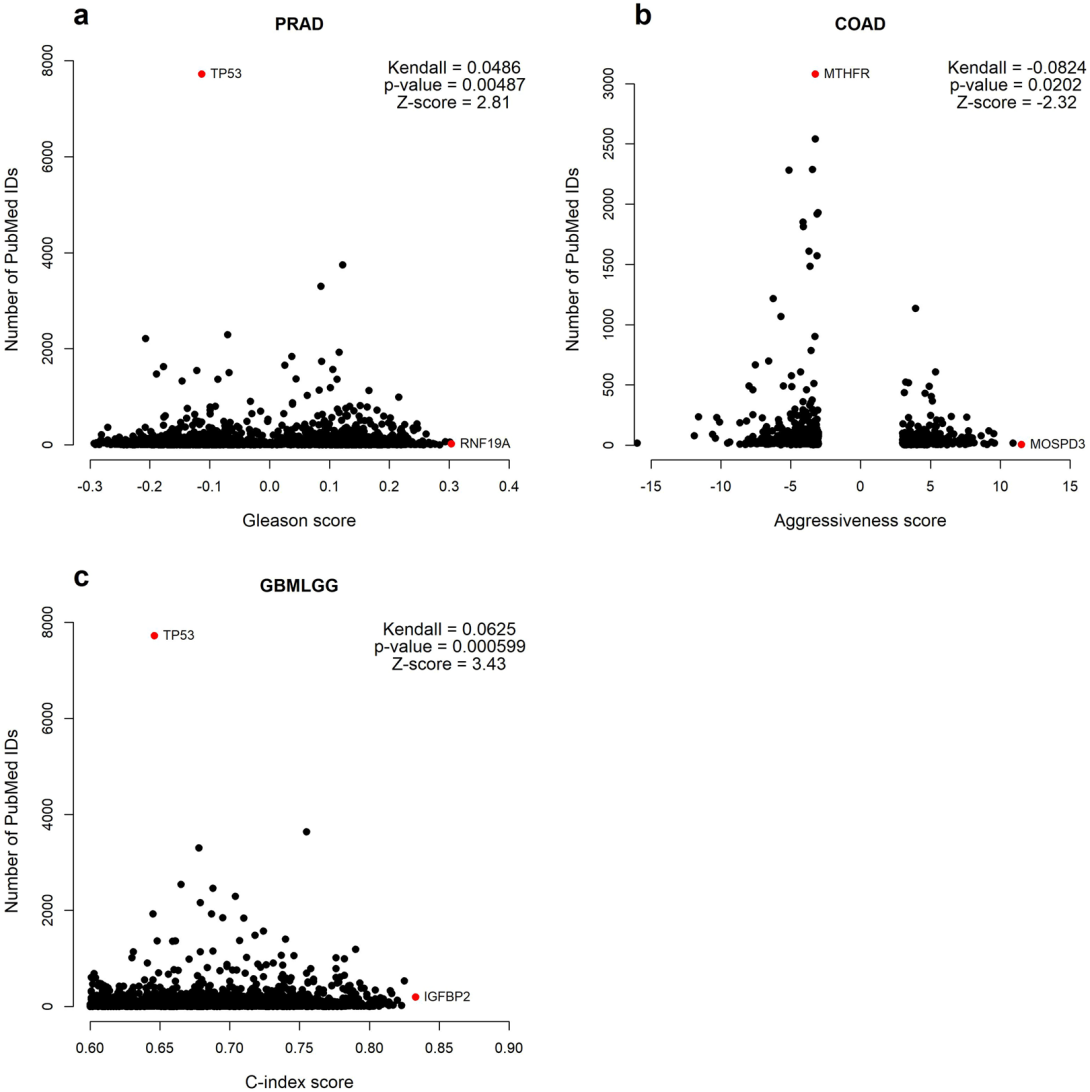
Kendall correlation between disease scores and number of PubMed publications for different cancers. (**a**) Gleason score correlated with PMIDs for prostate adenocarcinoma (PRAD) (**b**) aggressiveness score and PMIDS in colon adenocarcinoma (COAD) (**c**) C-index to number and PMIDs in glioma (GBMLGG). Outliers and genes with highest disease scores highlighted in red.

Additionally, when looking at a correlation between the number of PMIDs and the C-index in glioma, Gleason score, or colon cancer aggressiveness, there is again no consistent association between clinical phenotype metric and bibliometric interest (Fig. 3a–c), although this must be treated with caution because the statistical association of any given gene with a clinical outcome cannot be presumed to be directly equivalent to the magnitude of effect. Overall, however, the data indicate no reason to believe that research efforts have been focused on genes most obviously associated with clinical phenotype.

### Functionally enigmatic genes are not evenly distributed across the network

In order to examine whether the functionally enigmatic genes were equally distributed around the network, we took advantage of the WGCNA feature, which clusters genes into modules based on topological similarity (in essence, assuming that genes with similar “neighbors” are more likely to share a similar function). Of the total of 69 modules (32 modules for glioma, 20 modules for colon cancer, and 17 for prostate cancer), all but three modules (all in glioma) were significantly enriched for protein-protein interactions via STRING, indicating that overall WGCNA clustered together genes that were known to interact (Supplementary Table 2). While each module had a large fraction of functionally enigmatic genes, there was a substantial range - from a high of 80 percent to a low of 34 percent (Table 2, Prostate Dataset; other datasets shown in Supplementary Table 2). For each cancer dataset, the modules with the highest percentage of functionally enigmatic genes were enriched for terms associated with mRNA splicing, spliceosome, or ncRNA, while cell-cycle modules tended to have relatively few functionally enigmatic genes. This suggests that there are large areas of the “cancer map” - likely representing genes regulated in a coordinated way - where the overwhelming majority of genes have received scant attention in the literature. Even in the most studied module, 30 percent of the genes are comparatively understudied.

**Table 2:** Modules for the PRAD dataset ranked by percentage of functionally enigmatic genes.

PRAD Module Color	Total Genes	Functionally Enigmatic (%)	Unmapped in STRING (%)	PPI Enrichment p-value	GO Biological Process Term Description		
green	336	80.95	8	<1.00E-16	mRNA processing	mRNA splice site selection	spliceosomal complex assembly
cyan	60	80	4	6.35E-06	*translational initiation*	*mRNA export from nucleus*	*mRNA-containing ribonucleoprotein complex export from nucleus*
magenta	137	77.37	6	<1.00E-16	RNA splicing	mRNA processing	RNA processing
turquoise	3163	74.01	1	<1.00E-16	intracellular transport	single-organism intracellular transport	establishment of protein localization
yellow	823	71.81	1	<1.00E-16	single-organism intracellular transport	intracellular transport	neurogenesis
brown	974	70.12	1	<1.00E-16	chromatin modification	chromosome organization	peptidyl-lysine modification
salmon	72	69.45	4	4.28E-10	positive regulation of cellular protein metabolic process	inositol biosynthetic process	Golgi reassembly
blue	1451	67.26	1	<1.00E-16	cell morphogenesis involved in differentiation	extracellular matrix organization	extracellular structure organization
lightcyan	40	65	4	5.25E-14	muscle structure development	muscle filament sliding	actin-myosin filament sliding
grey60	40	62.5	2	<1.00E-16	defense response to virus	response to virus	type I interferon signaling pathway
red	285	62.11	0	1.75E-12	response to hormone	response to oxygen-containing compound	organonitrogen compound metabolic process
black	269	60.45	1	<1.00E-16	tissue development	epithelium development	cell adhesion
midnightblue	57	50.87	3	<1.00E-16	vasculature development	blood vessel development	angiogenesis
purple	127	50.39	1%	<1.00E-16	extracellular matrix organization	extracellular structure organization	collagen metabolic process
pink	234	43.59	2	<1.00E-16	immune response	defense response	positive regulation of immune system process
greenyellow	103	40.73	2%	<1.00E-16	response to organic cyclic compound	response to lipid	negative regulation of gene expression
tan	90	34.44	1%	<1.00E-16	cell cycle	mitotic cell cycle	cell cycle process

All modules were enriched for known protein-protein interactions within the STRING database, indicating that genes known to interact were grouped together. Top three GO Biological Process are shown; italics indicates enrichment for term was not significant at an FDR-corrected value of <0.05; all others were statistically significant. Full statistics are shown in Supplementary Table 2 along with other data sets.

**Figure 4:**
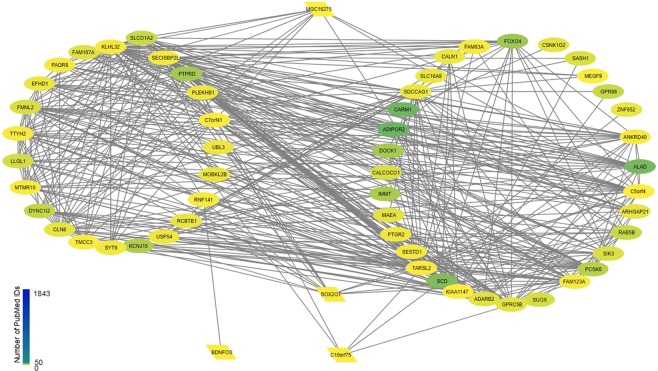
“Saddlebrown” module from the glioma dataset; genes predicted to be involved in CNS axon ensheathment are clustered together on the left, and other genes (many predicted to be involved in the phenylalanine metabolic process) are clustered on the right; ncRNA are shown in the middle. No gene had more than 150 PMIDs and the STRING database found no significant protein-protein interactions.

For the modules within the glioma dataset that were not enriched for known interactions, we investigated further to see if this might indicate plausible but as yet undiscovered functionally meaningful interactions. Two of the modules were highly enriched for genes on a specific chromosome position, likely reflecting chromosomal amplification and the biological significance is questionable. However, one module (“saddlebrown”) had no significant enrichment based on chromosome position, although it was associated with genes perturbed in several Gene Expression Omnibus (GEO) datasets related to glioblastoma and other diseases specfic to the central nervous system (CNS). We used the ARCHS4 database^19^ – which massively mines publicly available human and mouse RNA-Seq datasets to predict GO Biological Process – and selected the top five predicted categories for each gene. Despite the fact the CNS axon ensheathment has relatively few known genes in this category (33 total for humans), 24 of the 60 genes in the module were predicted to have this function (Fig. 4, Table 3). In addition, the remaining genes were predicted to be associated with phenylalanine metabolism or fatty acid elongation (Table 3). No prediction could be made for the four ncRNA, although interestingly, one of the ncRNA, c10orf75, which is also known as OLMALINC (Oligodendrocyte Maturation-Associated Long Intergenic Non-Coding RNA), is thought to be a primate-specific lncRNA that is involved the maintenance of oligodendrocyte maturation^20^. Within the network derived from the dataset, OLMALINC was connected to two genes predicted to be associated with myelination (KLH32 and PLEKHB1), one gene associated with metabolism (SCD), and a g-protein coupled receptor (GPRC5B). In each case the correlation was greater than 0.50 and highly statistically significant even after adjusting for tumor purity within glioma (Supplementary Table 5), although not in other cancers. Overall, this suggests that this module is indeed associated with myelination, and it would be a mistake to presume that the absence of known protein-protein interactions in the module indicates an artifact of the data or analysis methods, rather than simply unknown biology.

**Table 3:** Predicted GO terms for all genes in the “saddlebrown” module of the glioma dataset.

Term	Average Z-Score	Description	Genes in Module Predicted for GO Term	Total Number of Human Genes Annotated to GO Term
GO:0022010	7.58	central nervous system myelination	24	33
GO:0032291	5.47	axon ensheathment in central nervous system	24	33
GO:0016188	5.33	synaptic vesicle maturation	12	24
GO:0006559	6.09	L-phenylalanine catabolic process	12	23
GO:1902222	6.15	erythrose 4-phosphate/phosphoenolpyruvate family amino acid catabolic process	11	23
GO:0035641	5.40	locomotory exploration behavior	9	25
GO:0030497	6.24	fatty acid elongation	8	25
GO:0048172	5.20	regulation of short-term neuronal synaptic plasticity	7	27
GO:0071625	4.75	vocalization behavior	6	30
GO:0006558	6.36	L-phenylalanine metabolic process	6	11
GO:1902221	6.15	erythrose 4-phosphate/phosphoenolpyruvate family amino acid metabolic process	6	21
GO:2000463	4.78	positive regulation of excitatory postsynaptic potential	5	35
GO:0008366	5.46	axon ensheathment	5	145

ARCHS4 predictions were generated for each gene and the top five, ranked by z-score, were selected as possible annotations. Central nervous system myelination and phenylalanine catabolic process were predicted for 24 and 12 of the genes, respectively.

**Figure 5:**
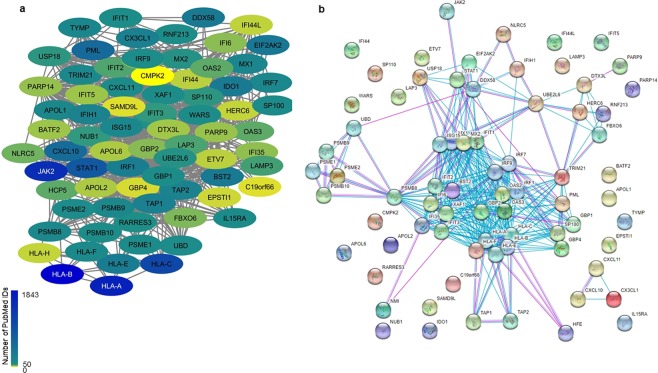
COAD “cyan” module APOL6 subnetwork. (**a**) Network derived from data showed STAT and APOL6 interacting, along with many other genes with relatively few PMIDs (indicated as node color). (**b**) PPI network from STRING, using experimental data (purple interactions), databases (blue) at medium confidence level. APOL6, and many of the other proteins, were not shown as connected to the STAT1 pathway.

Finally, to get a sense of the kind of biology likely missed, we have highlighted a handful of the genes that were functionally enigmatic. Within the COAD dataset network, APOL6 had the highest absolute ranking for aggressiveness, yet it has relatively few PMIDs, and was located in the “cyan” module annotated to defense response and the interferon signaling pathway. While most of the predicted interactions were verified in STRING, APOL6 was associated with multiple genes with relatively few PMIDs (Fig. 5). Based on the annotations of both the interacting genes and the resulting interaction network derived from STRING-DB, APOL6 appears to be associated with the STAT1 pathway, and indeed, APOL6 expression was strongly correlated with STAT1 in colon cancer (Spearman’s rank correlation 0.67, *p-*value 7.99e-54) and all other cancers with expression datasets greater than 400 patients after adjusting for tumor purity (Supplementary Table 4). Despite the relatively sparse literature base, the relevance to cancer is confirmed by one of the articles, which indicated that APOL6 has a role in mitochondrial-induced apoptosis in a colon cancer derived cell-line^21^.

**Figure 6:**
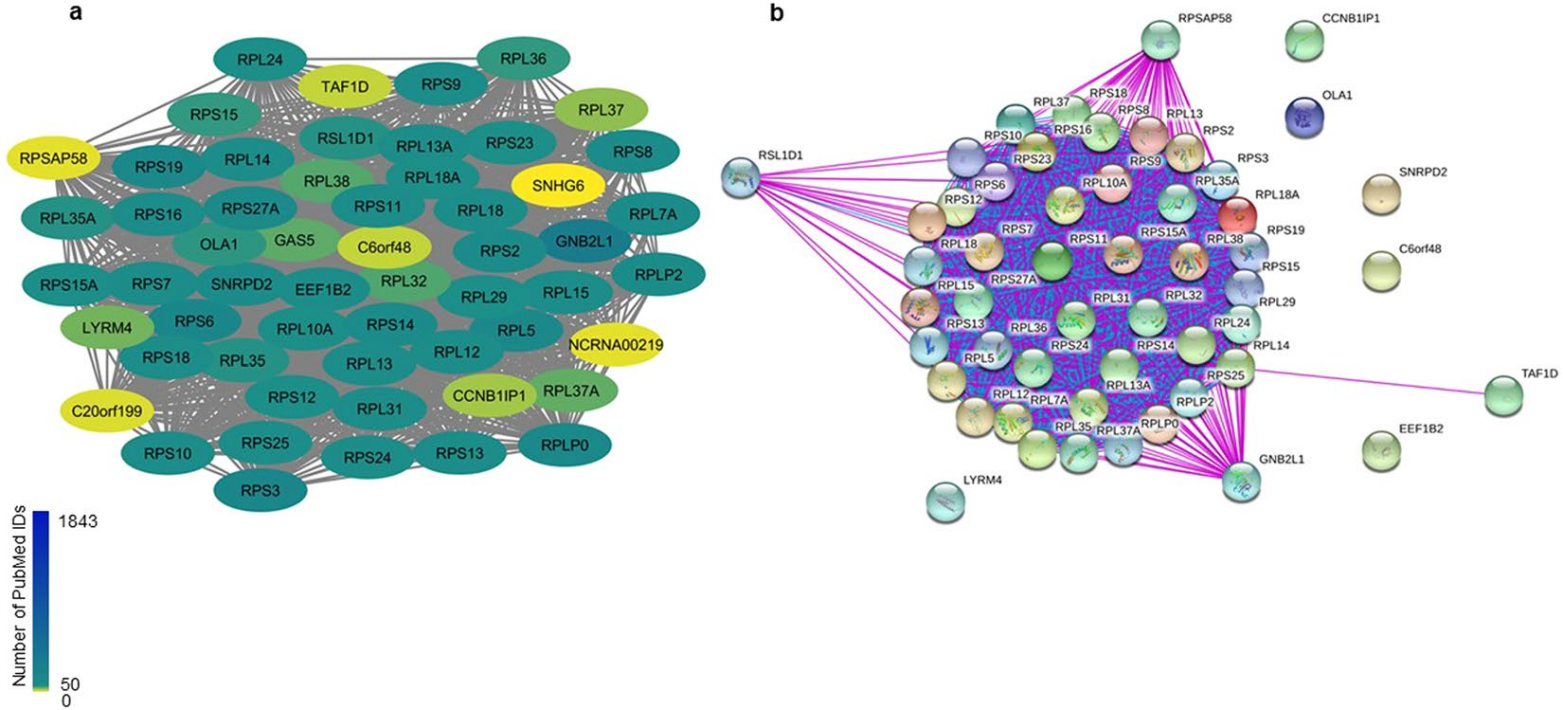
COAD C6orf48 subnetwork. (**a**) Network derived from data indicates that C6orf48 and GAS5 were correlated with several ribosomal protein subunits; nodes are colored according to PMID. None of the interacting genes had greater than 150 PMIDs. (**b**) Network derived from STRING database, experimental interactions in purple and database interactions in blue. Although the ribosomal subunits and most other proteins were known to interact, C6orf48 had no known connections, and GAS5 was unmapped in STRING.

Also within the COAD dataset, C6orf48 was the primate-specific gene with the highest absolute ranking for aggressiveness and was present in the glioblastoma and prostate networks, albeit it with a relatively low-scaled connectivity. C6orf48 - also known as SNHG32 (Small Nucleolar RNA Host Gene 32) - is an RNA gene and in the module (“blue”) it is associated with translation initiation, elongation or termination, and the only known homolog is in chimpanzee. Within the blue module, SNHG32 was consistently associated with several ribosomal proteins known to interact (Fig. 6), as well as with a comparatively well characterized long-noncoding RNA gene GAS5 (growth arrest specific 5). GAS5 is a known oncogene implicated in apoptosis^22^, and the correlation with C6orf48 and GAS5 was fairly strong in COAD (Spearman’s correlation 0.605; *p*-value 6.47e-42) and other datasets (Supplementary Table 3) after controlling for tumor purity. While the overall role of C6orf48/SNHGR2 remains murky, the association with both GAS5 as well as with ribosomal proteins subunits suggests the possibility that it acts to coordinate translation in response to GAS5-induced growth arrest.

Within the glioma dataset, two of the functionally enigmatic genes with a strong association with phenotype (MRS2 - mitochondrial and TOMM5, which has a primate-specific isoform) were in a module (“blue”) with the top three annotations as relatively high-level terms (single-organism intracellular transport, intracellular transport, and cellular catabolic process). Within the STRING database, there were no experimentally verified interactions between TOMM5 and MRS2 and the rest of the module, and relatively few when based on text-mining or co-expression. TOMM5 has only 11 publications, indicating that it is essential to the structural integrity of the mitochondrial outer membrane^23^. MRS2 is a magnesium transporter in the mitochondrial inner membrane, but the literature does offer clues as to why it might be a highly connected gene in the transcriptomic network: a knock-down *in vitro* study indicated that it acted to shift mitochondrial energy metabolism and was critical for cellular response to stress^24^; additionally, it was associated with cytochrome C release^25^ in gastric cancer cells, and therefore its connection to other mitochondrial genes and importance in cancer seems plausible.

## Discussion and Conclusions

The past few decades have seen an immense leap in our technological ability to interrogate the molecular processes of a cell. On top of this, there has been a profound improvement in both the quality and quantity of available high-throughput data, and this is most especially true for cancer, due to the concerted efforts of TCGA and others. It has been estimated that scientific output grows by approximately 10 percent every year^26^; PubMed has more than 27 million citations for biomedical and life science research literature^27^. Yet despite this, our analysis indicates that there remains a substantial portion of genes whose role in cancer is simply unknown or poorly characterized, about which the available literature is largely silent, and which we are unable to place with much conviction on a known pathway. In other words, cancer is being studied with a map of the cell that, for all practical purposes, has vast areas that are essentially unlabeled.

Additionally, it does not appear that our maps have been drawn by surveying the most important terrain. Our analysis is consistent with previous studies^15,28^ that have found no relationship between the relative biological importance of genes (as measured by connectivity in high-throughput data derived networks) and the literature dedicated to specific genes, suggesting instead that most genes, after their initial discovery, attract limited attention, while other genes attract disproportionate attention due, at least in part, to social trends and the tendency of the scientific community to be a “small-world”^28^. Our finding that gene importance (by any metric) has no substantive correlation with depth of literature would seem to indicate that even in well-studied diseases such as cancers - which have benefited from a wealth of -omics data - researchers tend to be more comfortable on familiar ground. This likely accounts for the recent finding that inequality of annotations between genes has in fact increased over time - that is genes follow a “rich-getting-richer” pattern - and that without a concerted effort, annotation bias will continue to significantly impede research^29^.

Indeed, this phenomenon may be more exaggerated in cancer, as research has generally focused on “driver” genes, where the mutation, methylation, or copy number variation confers a fitness-advantage, and most other alterations are presumed “passengers”^30^ (noise produced by the intrinsic instability of cancer). Nonetheless, focusing on the well-known and comparatively well-characterized “drivers” may miss much that is important for understanding how cancer responds to, or resists, therapy^30^, as many “passengers” may represent critical points in signaling networks that are mechanisms of invasiveness or drug-resistance. Just as increased data on mutations has revealed a “long molecular tail” of clinically significant mutations^31^, it seems likely that perturbed pathways within the cell have an equally long tail.

Clearly, the sheer volume of data available in projects such as TCGA means that leveraging existing knowledge to organize and analyze it is essential. At the same time, our results caution against any analysis that relies exclusively on annotations to make sense of the underlying biological importance of any group of genes. Our finding that functionally enigmatic genes are more likely to lack homologs in other species has an important consequence for clinical research, as it suggests that the areas where our map is inadequate will tend to be areas where rodent models will serve less well than data derived from human-based tissue. Other reasons for bias may simply reflect genes that are harder to study, and some genes - specifically ncRNA such as C6orf48 - are simply too new to have accumulated substantial knowledge. In addition, it is quite possible that some of the bias is due simply to the comparative difficulty of getting funding for more exploratory work. Our analysis has also shown that even when annotations are accurate and based on a well-understood biochemical function as well as on analogs (APOL6, MRS2), this can miss the appropriate context: the GO Annotations (lipid binding and magnesium transporter) were accurate, but failed to capture the broader context and therefore the likely relevance to cancer.

To be sure, any method of correlating genes will find both trivial correlations as well as biologically meaningful interactions, and any attempt to correlate gene signatures with phenotype will generate many false positives. This type of approach cannot ascertain either the true significance to cancer biology, or the ultimate molecular function, of any given gene. One significant limitation of our approach was that owing to the complexity and lack of an agreed-upon methodology for tumor deconvolution, we did not control for tumor purity in our network analysis, and this almost certainly disguised some pathways and over-emphasized others^32^. Many of the clinically significant genes were not present in the corresponding network either because their expression levels were low or because they lacked significant interactions with other genes, and no doubt some of these genes are indeed clinically and/or biologically relevant, but largely invisible with methods such as WGCNA. While WGCNA and other guilt-by-association approaches have proven a powerful way to uncover connections between genes in a manner not dependent on annotations^33,34^, it is important to remain mindful that like all methodologies, these approaches will have blind spots as well as spurious correlations. Nonetheless, our data indicate that several genes of significance have been overlooked in the literature, and this underscores the importance of taking data-driven approaches such as WGCNA, despite all the caveats. The ability of text-mining to effectively tease out connections among genes not yet placed on pathways will also likely be key to improving our maps by taking advantage of the available literature. Lastly, one key message is that when doing annotation-based analysis, it may be equally important to think about the absences, as many annotation tools provide no clear indication of how many genes lack annotations^35^. A researcher might get a statistically significant result based on a handful of genes and not realize that most genes queried have few, or low-quality, annotations available. Therefore, when doing any annotation or pathway-based analysis, it is perhaps equally important to think about the *absences* - for a scientist, the areas labelled *terra incognita* should be considered a challenge.

## Materials and Methods

### Data

Genes considered significant for unfavorable prognosis to cancer were downloaded from the Human Protein Pathology Atlas^36^, which used mRNA data from 17 different forms of human cancers. mRNA expression levels were correlated with clinical outcome for almost 8,000 cancer patients, and were considered significant when higher expression levels were associated with a worse outcome in any cancer via Kaplan-Meier survival plot with a p < 0.001^36^. For prostate and glioma, the RNA-Seq *vs*. clinical analysis from GDAC Firehose was used to correlate expression levels with endpoints; for glioma, significance was defined as a correlation with “days to death” with a *q*-value < 0.01 and C-Index greater than 0.6; for prostate cancer, significant genes were correlated +/− with Gleason score with a *q*-value <0.01. For colon cancer, genes were downloaded from the CRC Aggressiveness Explorer^37^, which is based upon a comprehensive TCGA analysis^17^, with an absolute value minimum cut-off of 3. Expression data for glioma (GBLMG), prostate cancer (PRAD), and colon cancer (COAD) were downloaded from the GDAC Firehose as normalized gene counts.

### Gene attributes

PubMed IDs (PMIDs) were identified by querying Entrez with the Entrez GeneID and getting a raw count of PMIDs that mapped to the genes; genes with less than 50 PMIDs were considered “functionally enigmatic”. GO annotation, GO Slim, and Panther pathways were identified using PantherDB^38^. Homologs and Human Phenotype Ontology were identified using EnrichR^39^. STRING DB^40^ was used for PPI enrichment, visualization of ontologies, and identifying experimental, text-mining, and co-expression relationships amongst proteins; all queries were done at 0.40 (medium) level of evidence. ARCHS4^19^ was used to predict GO Biological Process for genes.

### Networks

Networks were created using the WGCNA package^18^ by selecting the most variant 10,000 genes using median absolute deviance. Networks were based on Topological Overlap Metric and were created with a Pearson correlation raised to a soft β power of 6 based on scale-free topology criterion. Modules were assigned using a minimum module threshold of 30 and a height cut-off of 0.25. Scaled connectivity was calculated with the *fundamentalNetworkConcepts* function in the WGCNA package. Networks were visualized in Cytoscape^41^. Cross-cancer correlation for individual genes adjusted by tumor purity was done with TIMER^42^.

## Supplementary information

Supplementary Table 1, 3, 4, 5.

Supplementary Table 2.

## Data Availability

The datasets and computer codes produced in this study are available in the following databases: • Computer codes and data sets are available on GitHub (https://github.com/vy-p-tran/Functionally_enigmatic_genes). • RNA-seq data for PRAD, COAD, and GBMLGG datasets can also be downloaded from The Cancer Genome Atlas database (https://gdac.broadinstitute.org/).
