# Author Correction: Functionally Enigmatic Genes in Cancer: Using TCGA Data to Map the Limitations of Annotations

**DOI:** 10.1038/s41598-020-66767-3

**Published:** 2020-06-11

**Authors:** Alexandra Maertens, Vy P. Tran, Mikhail Maertens, Andre Kleensang, Thomas H. Luechtefeld, Thomas Hartung, Channing J. Paller

**Affiliations:** 1 Johns Hopkins University, Bloomberg School of Public Health, Center for Alternatives to Animal Testing (CAAT), Baltimore, MD United States; 2 ToxTrack LLC, Baltimore, USA; 3 University of Konstanz, CAAT-Europe, Konstanz, Germany; 4 Johns Hopkins University, Oncology, Baltimore USA

Correction to: *Scientific Reports* 10.1038/s41598-020-60456-x, published online 05 March 2020

The original version of this Article contained a typographical error in the spelling of the author Vy P. Tran, which was incorrectly given as Vy H. Tran.

As a result, within the Acknowledgements section:

“V.H.T. is supported by NIH training grant number 2T32ES007141. C.P. is supported by NIH grant numbers P30CA006973 and K23 CA197526.”

now reads:

“V.P.T. is supported by NIH training grant number 2T32ES007141. C.P. is supported by NIH grant numbers P30CA006973 and K23 CA197526.”

Finally, the Author Contributions section:

“Conceptualization, A.M., C.P.; Methodology, A.M., T.H.L., V.H.T.; Formal Analysis, A.M., V.H.T., T.H.L., M.M., A.K.; Writing—Review & Editing, A.M., V.H.T., C.P., T.H.; Visualization, V.H.T., A.M.; Supervision: C.P., T.H.”

now reads:

“Conceptualization, A.M., C.P.; Methodology, A.M., T.H.L., V.P.T.; Formal Analysis, A.M., V.P.T., T.H.L., M.M., A.K.; Writing—Review & Editing, A.M., V.P.T., C.P., T.H.; Visualization, V.P.T., A.M.; Supervision: C.P., T.H.”

This has now been corrected in the PDF and HTML versions of the Article.

